# Composition and Chemical Variability of Trunk Bark Oil of *Pachylobus klaineanus* (Pierre) Guillaumin. From Côte D'Ivoire

**DOI:** 10.1002/cbdv.71670

**Published:** 2026-09-03

**Authors:** Stéphane Gervais Gooré, Bi Tra Marc Gabin Djié, Bi Goblé Landry Guessan, Zana Adama Ouattara, Thomas Maroselli, Yves‐Alain Békro, Mathieu Paoli, Félix Tomi

**Affiliations:** ^1^ UFR Sciences De la Mer Université Polytechnique de San‐Pedro San Pedro Côte d'Ivoire; ^2^ Laboratoire de Chimie Bio‐Organique et de Substances Naturelles (LCBOSN) UFR SFA Université Nangui Abrogoua Abidjan Côte d'Ivoire; ^3^ Université de Corse‐CNRS UMR 6134 SPE Équipe Chimie et Biomasse Ajaccio France

**Keywords:** chemical variability, Côte d'Ivoire, essential oil composition, *Pachylobus klaineanus* (Pierre) Guillaumin, trunk bark

## Abstract

The chemical composition of 50 individual trunk bark oil samples from plants of *Pachylobus klaineanus* (Pierre) Guillaumin. collected in eight Ivoirian localities was investigated by GC‐FID (50 samples), GC/MS (30 samples), and ^13^C‐NMR (23 samples). Eighty‐three compounds were identified. Oil samples are dominated by monoterpenes. The main components were α‐pinene (28.8%–76.2%), β‐pinene (2.0%–46.3%), limonene (0.9%–13.8%), *p*‐cymene (0%–19.2%), and myrcene (0%–17.9%). The 50 oil compositions were submitted to hierarchical clustering and principal components analyses, which allowed the distinction of two groups within the oil samples. Group II could be divided into two subgroups, IIa and IIb. Group I (23 samples) was characterized by the pre‐eminence of α‐pinene; subgroup IIa (19 samples) was dominated by α‐pinene followed by β‐pinene, while IIB (8 samples) is represented by α‐pinene and β‐pinene in association with monoterpene hydrocarbons (limonene and *p*‐cymene). The trunk bark essential oil of this species was reported for the first time.

## Graphical abstract

Composition of trunk bark oil of *Pachylobus klaineanus*.

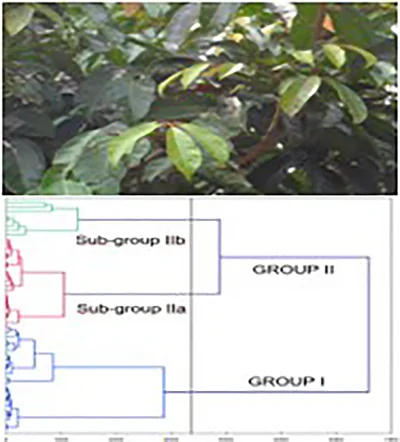

## 1 Introduction

*Pachylobus klaineanus* (Pierre) Guillaumin. (commonly known as monkey plum, African cherry fruit or also known as *Dacryodes klaineana (Pierre)* H.J. Lam. or *Pachylobus klaineana* (Pierre) Oliv.) belongs to Burseraceae family [1, 2]. A lot of synonyms are reported for *P. klaineanus*, that is, *Sorindeia deliciosa, Santiriopsis klaineana*, or *Haematostaphis delicioca*. The taxonomic status of the species *Aucoumea klaineana*, which has sometimes been treated as synonym of *Dacryodes klaineana* remains unclear. The genus includes about 70 species present in the tropical regions of America, Asia, and Africa [3]. In Africa, around forty species have been identified [4]. *P. klaineanus* is a medium‐sized tree up to 25 m high and 60 cm in diameter. Its shaft is devoid of branches for 10 m. The outer bark is greenish‐gray or blackish, very scaly while the inner bark is pinkish‐brown or reddish‐brown, with a turpentine smell. The leaves are alternate, composed, imparipinnate with three pairs of leaflets (length: 15–30 cm), covered with reddish pubescence when young, glabrous but with persistent hairs on the petiole, rachis and midrib. The inflorescences are terminal or sometimes axillary panicles (length: 10–22 cm), covered with dense reddish pubescence. Its flowers are unisexual and regular, with pedicels measuring from 2 to 4 mm in length. The fruits are ovoid drupes (length: about 2 cm; width: 1.5 cm) and orange when ripe, containing fleshy, fragrant pulp. The plant is traditionally used in Côte d'Ivoire to treat tachycardia and cough [5]. In the southeast of the country, the leaves, crushed with those of *Chlamydocola chlamydantha* and *Musanga cecropioïdes*, are used by the Akyés people to relieve women suffering from painful menstruation [6]. Boiled roots of *P. klaineanus* have traditionally been used to treat skin disease in Akwa Ibom State of Nigeria [7]. Its wood is used in construction and for mortars, axe handles and wagons. It has also been recommended for telegraph poles and railway sleepers. It is considered suitable for interior joinery, furniture and parquetry and has been used for paper making [8]. The fruit is eaten raw or cooked and the pulp is boiled or roasted to yield a kind of butter.

Various phytochemical investigations on *P. klaineanus* and *related synonyms (D. klaineana, A. klaineana)* have revealed, on one hand, the presence of glycosides, flavonoids, saponins, tannins, proteins, steroids, terpenoids, alkaloids in stem bark [9] and, on the other hand, the presence of tannins, flavonoids, alkaloids and cardiac glycosides in the kernel [10] and terpenoids in the pulp [11]. The only studies on the chemical composition of essential oil from this species concern the resin. *p*‐Cymene (30.2%), α‐pinene (20.6%) and α‐phellandrene (11.2%), δ‐3‐carene (72.31%) and terpinolene (6.28%) in Gabon [12, 13]; α‐phellandrene (30.9%), α‐pinene (29.3%), *p*‐cymene (9.2%) and 1,8‐cineole (9.0%) in Cameroon [14] were the major components.

This study is part of our work on the chemical characterization of essential oils from Côte d'Ivoire [15, 16, 17, 18, 19]. The aim of the present work was to i) collect a large number of plant material harvested on 50 individual plants growing wild in eight locations of Côte d'Ivoire; ii) prepare the essential oil (hydrodistillation); iii) perform chemical analysis (GC‐FID, GC/MS and ^13^C‐NMR); iv) submit the 50 compositions to statistical analysis in order to evidence homogeneity or chemical variability; and iv) finally characterize, for the first time, trunk bark essential oil of *P. klaineanus*.

## 2 Results and Discussion

**FIGURE 1:**
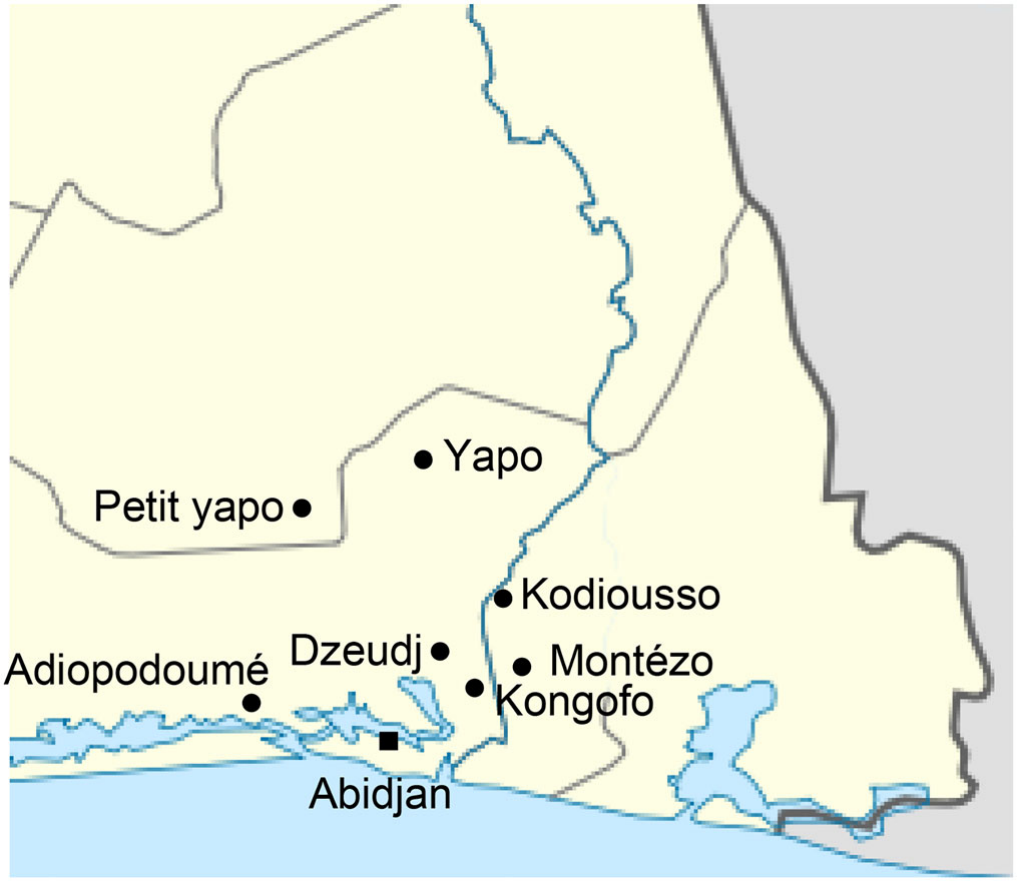
Localities of harvest of trunk bark of *P. klaineanus* from Côte d'Ivoire.

In total, 50 oil samples have been isolated from trunk bark of *P. klaineanus* harvested in eight localities of Côte d'Ivoire (Figure 1). Yields ranged from 0.02% to 0.13% (w/w based on dried material). The samples were analyzed by GC‐FID (50 samples), GC/MS (30 samples) and then by ^13^C‐NMR (23 samples) according to a computerized method developed at the University of Corsica that allowed identification of individual components in the mixture at content as low as 0.4%–0.5%. Four samples are selected and chosen on the basis of their chromatographic profiles to provide a representation overview of the chemical diversity encountered. Then, we studied the chemical variability of all oil samples by statistical analysis.

### 2.1 Analysis of Four Essential Oil Selected Samples

Detailed analysis of four trunk bark samples (Ad1, Ad2, Dz28, and Ko48) carried out by the combination of GC‐FID on two columns of different polarity, GC/MS and ^13^C‐NMR (Table 1) [20, 21] enabled the identification of 62 compounds, representing, respectively, 92.8%, 96.7%, 96.1%, and 85.0% of the whole composition of the samples. All samples are mainly composed of monoterpenes (68.4%–92.9%) compared to sesquiterpenes (3.2%–24.4%). α‐Pinene was always the major compound (27.9%–38.8%). The second most important compound is β‐pinene (14.7%–22.3%) in three samples (Ad2, Dz28, and Ko48) and myrcene (17.9%) in sample Ad1. Limonene occurred in samples Dz28 and Ko48 at very significant levels (13.8% and 11.8%, respectively) and was practically absent in the others (0.9% in sample Ad1 and 2.9% in sample 2). Terpinolene is present in sample Dz28 at 11.4% and does not exceed 0.7% in the other three. Several sesquiterpenes are present in appreciable level but in a more sporadic manner: cyperene (up to 5.1%, sample Ko48), rotundene (up to 4.2%, sample 48), α‐selinene (up to 4.2% in sample 50), β‐selinene (up to 4.7% in sample 50), and t‐cadinol (up to 5.1% in sample 1). Finally, α‐gurjunene was present only in sample Ad1 with a content of 3.1%.

**TABLE 1:** Components of four samples of *P. klaineanus* trunk bark oil.

	Components^a^	RIa^b^	RIp^c^	Ad1	Ad2	Dz28	Ko48	Identification mode
1	α‐Thujene	921	1017	0.5	0.9	1.6	0.4	RI, MS, ^13^C‐NMR
2	α‐Pinene	929	1 017	36.3	38.8	35.5	27.9	RI, MS, ^13^C‐NMR
3	α‐Fenchene	940	1 055	tr	0.1	0.1	tr	RI, MS
4	Camphene	942	1 065	0.2	0.6	0.7	0.4	RI, MS, ^13^C‐NMR
5	Thuja‐2,4(10)‐diene	945	1 128	0.1	—	0.1	tr	RI, MS
6	Sabinene	964	1 123	0.2	0.5	0.3	0.1	RI, MS
7	β‐Pinene	969	1 113	5.5	22.3	20.1	14.7	RI, MS, ^13^C‐NMR
8	Myrcene	979	1 161	17.9	1.1	1.9	1.6	RI, MS, ^13^C‐NMR
9	α‐Phellandrene	995	1 166	—	0.1	0.2	8.2	RI, MS, ^13^C‐NMR
10	δ‐3‐Carene	1 003	1 148	0.1	—	—	—	RI, MS
11	α‐Terpinene	1 007	1 182	—	0.4	0.3	0.2	RI, MS
12	*p*‐Cymene	1 010	1 272	1.0	0.5	0.9	2.3	RI, MS, ^13^C‐NMR
13	Limonene*	1 019	1 201	0.9	2.7	13.8	11.8	RI, MS, ^13^C‐NMR
14	β‐Phellandrene*	1 019	1 211	0.6	1.3	0.6	3.1	RI, MS, ^13^C‐NMR
15	(*Z*)‐β‐Ocimene	1 024	1 233	0.4	0.1	—	—	RI, MS
16	(*E*)‐β‐Ocimene	1 035	1 250	2.9	1.5	tr	—	RI, MS, ^13^C‐NMR
17	γ‐Terpinene	1 047	1 245	tr	0.9	0.4	0.3	RI, MS, ^13^C‐NMR
18	*p*‐Cymenene	1 071	1 439	0.1	0.1	1.0	tr	RI, MS, ^13^C‐NMR
19	Terpinolene	1 077	1 283	0.1	0.7	11.4	0.5	RI, MS, ^13^C‐NMR
20	Linalool	1 082	1 545	—	0.1	—	—	RI, MS
21	Fenchol	1 096	1 581	—	0.1	0.1	0.1	RI, MS
22	*trans*‐Pinocarveol	1 121	1 655	0.3	0.2	0.2	—	RI, MS
23	*p*‐Cymen‐8‐ol	1 157	1 849	0.1	—	1.0	—	RI, MS, ^13^C‐NMR
24	Terpinen‐4‐ol	1 160	1 601	0.4	1.0	0.8	0.3	RI, MS, ^13^C‐NMR
25	Myrtenal	1 167	1 628	0.3	0.2	0.3	—	RI, MS
26	α‐Terpineol	1 170	1 696	0.5	2.3	1.4	1.0	RI, MS, ^13^C‐NMR
27	Verbenone	1 178	1 707	—	—	0.2	—	RI, MS
28	α‐Cubebene	1 348	1 456	0.4	0.1	—	0.1	RI, MS
29	Cyclosativene	1 366	1 479	0.2	—	—	0.1	RI, MS
30	α‐Ylangene	1 369	1 481	—	0.2	—	—	RI, MS
31	α‐Copaene	1 375	1 490	1.2	2.1	0.1	0.5	RI, MS, ^13^C‐NMR
32	β‐Elemene	1 385	1 587	0.4	0.5	—	—	RI, MS
33	Cyperene	1 398	1 523	0.2	0.1	1.2	5.1	RI, MS, ^13^C‐NMR
34	α‐Gurjunene	1 408	1 525	3.1	—	—	—	RI, MS, ^13^C‐NMR
35	*cis*‐α‐Bergamotene	1 409	1 566	—	1.1	—	—	RI, MS, ^13^C‐NMR
36	α‐Santalene	1 415	1 568	0.1	—	—	—	RI, MS
37	(*E*)‐β‐Caryophyllene	1 416	1 593	0.7	1.0	0.1	—	RI, MS
38	β‐Copaene	1 424	1 587	0.1	—	—	—	RI, MS
39	Aromadendrene	1 435	1 602	1.1	—	—	—	RI, MS, ^13^C‐NMR
40	Spirolepechinene	1 444	1 643	—	—	—	2.0	RI, MS, ^13^C‐NMR
41	α‐Humulene	1 448	1 665	0.8	0.4	—	—	RI, MS, ^13^C‐NMR
42	Rotundene	1 455	1 634	—	—	0.9	4.2	RI, MS, ^13^C‐NMR
43	*allo*‐Aromadendrene	1 456	1 640	1.7	0.6	0.1	—	RI, MS, ^13^C‐NMR
44	γ‐Muurolene	1 468	1 683	0.1	0.1	—	—	RI, MS
45	Germacrene D	1 474	1 704	—	0.6	—	—	RI, MS
46	γ‐Humulene	1 475	1 718	—	2.1	—	—	RI, MS, ^13^C‐NMR
47	β‐Selinene	1 479	1 713	0.4	2.3	—	—	RI, MS, ^13^C‐NMR
48	α‐Selinene	1 489	1 720	—	1.6	—	—	RI, MS, ^13^C‐NMR
49	α‐Muurolene	1 491	1 720	0.2	—	—	—	RI, MS
50	γ‐Cadinene	1 503	1 755	2.6	1.7	0.1	—	RI, MS, ^13^C‐NMR
51	*trans*‐Calamenene	1 507	1 829	1.4	—	0.1	—	RI, MS, ^13^C‐NMR
52	δ‐Cadinene	1 513	1 752	0.7	1.4	0.2	—	RI, MS, ^13^C‐NMR
53	(*E*)‐Nerolidol	1 546	2 037	0.8	—	—	—	RI, MS, ^13^C‐NMR
54	Spathulenol	1 561	2 117	0.7	—	0.1	—	RI, MS, ^13^C‐NMR
55	Caryophyllene oxide	1 566	1 978	0.3	0.1	—	—	RI, MS
56	Ledol	1 589	2 021	0.5	—	—	—	RI, MS
57	Humulene oxide II	1 590	2 031	0.3	—	—	0.1	RI, MS
58	1,10‐Di‐e*pi*‐Cubenol	1 599	2 054	0.3	0.3	—	—	RI, MS
59	τ‐Cadinol	1 622	2 165	5.1	3.9	0.3	—	RI, MS, ^13^C‐NMR
60	β‐Eudesmol	1 631	2 224	0.5	—	—	—	RI, MS, ^13^C‐NMR
61	α‐Cadinol	1 635	2 226	0.2	—	—	—	RI, MS
62	α‐Eudesmol	1 636	2 215	0.3	—	—	—	RI, MS
	Total			92.8	96.7	96.1	85.0	
	Yield			0.03	0.02	0.05	0.02	
	Monoterpene hydrocarbons			66.8	72.6	88.9	71.5	
	Oxygenated monoterpenes			1.6	3.9	4.0	1.4	
	Sesquiterpene hydrocarbons			15.4	15.9	2.8	12.0	
	Oxygenated sesquiterpenes			9.0	4.3	0.4	0.1	

^a^ Order of elution and percentages of individual components are given on apolar column except for compounds marked with an asterisk (polar column).

^b^ RIa, retention indices determined on the apolar column (BP‐1).

^c^ RIp, retention indices determined on the polar column (BP‐20).

Abbreviation: tr, trace level (< 0.05%).

Special attention was needed for the identification of spirolepechinene (2% in sample Ko48). In fact, the MS spectrum of this compound was not compiled in MS libraries at our disposal and in our home‐made MS library. The chemical shifts of the carbon atoms of this compound, recorded on the NMR spectrum of sample Ko48, correspond to those described in the literature [22]. Despite the relatively low content of the compound (2.0%), all signals, including those of the quaternary carbons, were detected. The DEPT 135 sequence performed on the mixture confirmed the multiplicity of all the carbon atoms in the molecule. Furthermore, the retention index on a non‐polar column for this sesquiterpene (IR = 1444) remains consistent with that obtained on an HP‐5 MS capillary column (IR = 1450) [23].

The chemical composition of trunk bark essential oil of *P. klaineanus* differed from that of resins collected in Gabon, which are dominated by *p*‐cymene (30.2%), α‐pinene (20.6%) and α‐phellandrene (11.2%) [12] and δ‐3‐carene (72.31%) and terpinolene (6.28%) [13], as well as that of resin from Cameroon, whose main components were α‐phellandrene (30.9%), α‐pinene (29.3%), *p*‐cymene (9.2%), and 1,8‐cineole (9.0%) [14]. Significant differences are also observed between the composition of trunk bark essential oil of *P. klaineanus* and that of different species of the genus *Pachylobus*. Indeed, the major constituents from the resin oil from *D. edulis* growing in Gabon were sabinene (21.76%), terpinen‐4‐ol (19.79%), α‐pinene (17.47%), and *p*‐cymene (11.29%) [24]. Fruits essential oil from *D. peruviana* in the Ecuadorian Amazon is characterized by α‐phellandrene (50.32%–52.35 %), limonene (23.03%–22.51%), and α‐pinene (8.27%–8.45%)  [25, 26]. In Cameroon, various parts of *D. edulis* have been investigated: Essential oil from the resin contained *p*‐cymene (30.32%), α‐thujene (28.58%), α‐phellandrene (27.14%) and β‐phellandrene (10.16%) as the main components; stem barks essential oil were dominated by *p*‐cymene (35.14%), *trans*‐carveol (22.60%), α‐thujene (14.86%) and β‐phellandrene; leaf essential oil was characterized by elemol (29.22%), caryophyllene oxide (15.26%) and *trans*‐carveol (11.80%) as major components [27]. Leaf oil of the same species from Congo‐Brazzaville contained mostly sesquiterpenes: cadinol (1%–4%), β‐caryophyllene (3%–10%), *cis*‐bergamotene (tr–11%), *trans*‐bergamotene (2%–11%), sesquisabinene (9%–11%) and eremophylene (1%–9%) [28]. Myrcene (45.3%) and α‐pinene (8.8%) were the dominant constituents of the fruit oil and β‐caryophyllene (26.4%) of the leaf oils. The stem‐bark essential oil contained predominantly terpinen‐4‐ol (25.6%) and limonene (12.5%) and a mixture of α‐thujene and α‐pinene (25.2%), whilst α‐phellandrene (26.5%), limonene (10.2%) and β‐pinene (8.7%) were the major component of the root‐bark oil of the Gabonese species [29].

Although dominated by monoterpenes, the notable differences between the chemical compositions of the four samples investigated suggested variability in the composition of trunk bark essential oil.

### 2.2 Chemical Variability of *P. klaineanus* Trunk Bark Oil

Fifty oil samples of *P. klaineanus* have been isolated from trunk bark of individual trees collected in eight Ivoirian southeast localities (Figure 1). Trunk barks were dried for three days before essential oils extraction. Yields were in the range of 0.02%–0.13% (w/w vs. dried material). All the oil samples were analyzed by GC (in combination with retention indices, GC‐FID measured on two columns of different polarity) (Table S1). On the basis of their chromatographic profile, thirty oil samples were also analyzed by GC‐MS and twenty‐three oil samples have been subjected to ^13^C‐NMR.

The combination of the chromatographic and spectroscopic techniques has allowed the identification of 83 compounds accounting for 83.3%–99.1% of the total composition of the samples. All oil samples are mainly rich in monoterpenes compounds (46.6%–96.0%) compared to sesquiterpenes compounds (0.4%–38.6%). The contents of the main components are reported in Table 2 (compounds present at least once at more than 1%).

**TABLE 2:** Mean in samples of Group I and Sub‐Group IIa and IIb percentages, and standard deviations of the main components of Ivoirian *P. klaineanus* trunk bark oil samples.

Groups/sub‐groups	Group I				Sub‐group IIa				Sub‐group IIb			
Number of oil samples	23				19				8			
**Components**	M	SD	Min	Max	M	SD	Min	Max	M	SD	Min	Max
α‐Thujene	1.7	1.5	0.2	6.3	1.8	1.3	0.5	5.5	2.1	2.8	0.3	8.6
α‐Pinene	54.2	12.9	28.8	76.2	44.5	6.0	32.9	56.2	23.4	10.2	3.0	35.5
β‐Pinene	6.6	2.0	3.9	11.4	26.2	8.3	13.7	46.3	15.0	9.8	2.0	30.2
Myrcene	2.5	6.1	0.0	24.6	1.3	1.8	0.1	7.6	1.0	0.6	0.1	1.9
α‐Phellandrene	0.1	0.1	0.0	0.7	0.1	0.1	0.0	0.3	4.1	7.6	0.0	19.8
*p*‐Cymene	2.5	2.8	0.2	11.7	1.4	1.2	0.0	3.9	6.4	7.3	0.9	21.4
Limonene	3.0	2.7	0.9	11.2	2.9	2.4	1.4	11.9	9.6	3.6	3.5	13.8
Terpinolene	1.7	3.6	0.0	15.0	0.8	1.0	0.0	4.6	5.6	7.2	0.3	19.2
Terpinen‐4‐ol	1.8	2.2	0.1	7.9	1.4	1.3	0.2	4.8	3.4	5.3	0.0	16.1
α‐Terpineol	1.3	1.0	0.2	4.9	2.2	1.8	0.6	8.5	2.0	1.4	0.6	4.8
cis‐α‐Bergamotene	0.2	0.3	0.0	0.8	0.1	0.3	0.0	1.1	8.6	14.8	0.0	25.7
τ‐Cadinol	2.6	2.5	0.0	8.9	2.1	2.0	0.0	5.8	0.3	0.1	0.2	0.4
α‐Cadinol	0.2	0.2	0.0	0.5	0.0	0.0	0.0	0.0	0.4	0.2	0.2	0.6

*Note*: Percentages are given on apolar column (BP‐1).

Abbreviations: M, mean; Max, maximum content; Min, minimum content; SD, standard deviation.

**FIGURE 2:**
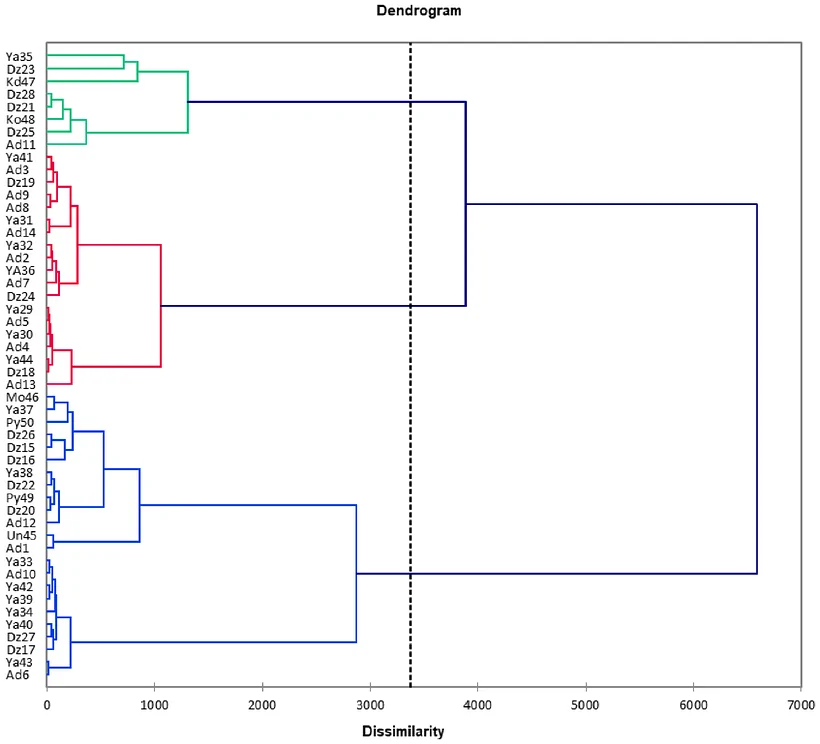
Hierarchical cluster analysis of the compositions of 50 *P. klaineanus* essential oils.

**FIGURE 3:**
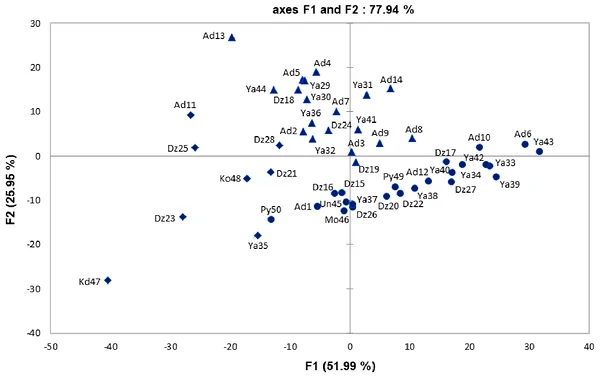
Discriminant analysis scatterplot of the oil constituents from 50 *P. klaineanus* essential oils.

The existence of two groups (I, II), based on the major components, was suggested by the hierarchical cluster analysis (HCA; dendrogram: Figure 2). The second group was subdivided into two subgroups, IIa and IIb (dashed line, Figure 2). Conversely, the discrimination is less evident by principal component analysis (PCA, plot shown in Figure 3), for which the plan defined by the first two axes described 77.94% of the total variance of the populations.

**FIGURE 4:**
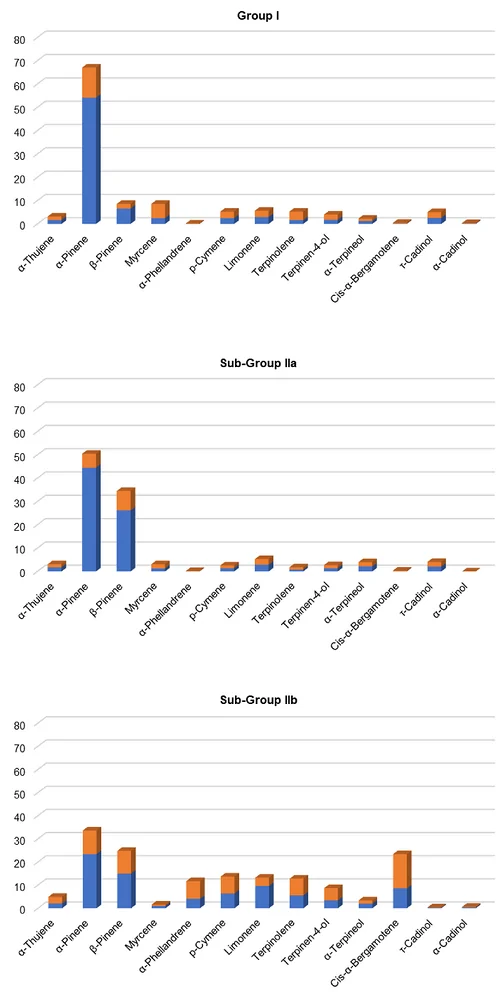
Contents of the major components of *P. Klainean*us trunk bark oil samples of Group I and Sub‐groups IIa and IIb. Blue, mean percentage; brown, standard deviation.

The discriminant components were visualized as vectors on the biplot graph and reported in Table 2. They are also represented as bar chats in Figure 4. Group I (23 samples) was characterized by α‐pinene with a mean value of 54.2% (SD = 12.9%). Group IIa (19 samples) is dominated by the association α‐pinene/β‐pinene (M = 44.5%; SD = 6.0)/ /M = 26.2%; SD = 8.3). The sub‐groups IIa (19 samples) and IIb (8 samples) were discriminated on the basis of pinene contents (α‐pinene: *M* = 44.5% vs. 23.4%; β‐pinene: *M* = 26.2% vs. 15.2%) and monoterpenes hydrocarbons contents limonene (*M* = 2.9% vs. 9.6%) and *p*‐cymene (*M* = 1.4% vs. 6.4%). This sub‐group IIb appears less homogeneous than group I and subgroup IIa. Thus, the percentage of cis‐α‐Bergamotene can reach 25.7% in this sub‐group.

There is no correlation between the locations and the chemical compositions. In fact, the three types of chemical composition are found in three localities, Adiopodoumé (littoral,14 samples), Dzeudji (littoral, 14 samples) and Yapo abbé (inland, 16 samples), leading to important intra‐station variability. In Dzeudji and Yapo abbé stations, groups I and II are represented in equal proportions: 7/7 for Dzeudji and 8/8 for Yapo abbé. In contrast, at the Adiopodoumé station, Group II is approximately twice as abundant as Group I, that is, 10 versus 4 samples. It is noticeable that subgroup IIb, although comprising only eight samples, is more common in the Dzeudji location: 4 samples. It could be pointed out that all the harvesting sites belonged to the same climate zone (equatorial humid) and were subjected to the same environmental conditions.

## 3 Conclusion

Essential oils from trunk bark of *P. klaineanus* have been analyzed, for the first time, by a combination of chromatographic and spectroscopic techniques. The detailed analysis of four selected samples has shown that oils were rich in monoterpenes compounds and mainly dominated by α‐ and β‐pinenes. Besides, to evidence chemical variability, on the basis of chromatographic profiles, 50 oil compositions were submitted to statistical analysis. Hierarchical cluster analysis led to the occurrence of two groups of compositions within the trunk bark oil, more or less confirmed by principal component analysis. α‐pinene was, by far, the major component of the oil samples of Group I; subgroup IIa was characterized by both α‐pinene and β‐pinene, while the composition of the samples of Group IIb was dominated by α‐pinene and β‐pinene accompanied by limonene, *p*‐cymene or terpinolene. According to the present results, there is no correlation between the location of harvest (inland and littoral) and the chemical composition.

## 4 Experimental Section

### 4.1 Plant Material

Trunk bark from 50 individual *P. klaineanus* trees have been harvested in eight Ivoirian localities: Adiopodoumé (samples Ad1–Ad14; 5°20′27.08″N–4°8′0.47″O), Dzeudji (samples Dz15–Dz28; 5°35′42.37″N–3°51′5.52″O), Yapo abbé (samples Ya29–Ya44), University Nangui Abrogoua, Abidjan (sample Un45; 5°23′21.32″N–4°1′8.78″O), Montézo (sample Mo46;5°29′4.29″N–3°44′9.21″O), Kodioussou (sample Kd47; 5°45′45.96″N–3°46′8.57″O), Kongofon (sample Ko48; 5°27′20.64″N–3°51′1.47″O) and Petit yapo (samples Py49‐Py50; 5°47′51″N –4°8′21″O) from August to November 2021. The sampling was done considering the phenological stage (full flowering stage) of different locations (inland and littoral locations). Plant material was authenticated by Mr Assi Jean, technician at the Herbarium of the Centre National de Floristique (Abidjan, Côte d'Ivoire), and Mr Téhé Henry from the Centre Suisse de Recherches Scientifiques (Abidjan, Côte d'Ivoire) by comparison with several voucher specimens collected from different forests that are available at the Centre National de Floristique. A voucher specimen has been deposited with the herbarium of the Centre National de Floristique (CNF) under the reference LAA 13121.

### 4.2 Oil Distillation

Essential oil (EO) samples were obtained by hydrodistillation from the three‐day‐dried trunk bark (1,200–1,850 g) with a Clevenger‐type apparatus during 3.5 h. Oil samples were dried over anhydrous sodium sulphate (Na_2_SO_4_) and stored in a freezer until analysis. Yields have been calculated from dried material (w/w).

### 4.3 Analytical GC

GC analyses were done on a Perkin‐Elmer Clarus 500 gas chromatograph (FID) equipped with two fused silica capillary columns (50m × 0.22mm, 0.25 µm film thickness), BP‐1 (polydimethyl siloxane) and BP‐20 (polyethylene glycol). The oven temperature was programmed from 60°C to 220°C at 2°C/min and then held isothermal at 220°C for 20 min; injector temperature: 250°C; detector temperature: 250°C; carrier gas: hydrogen (1.0 mL/min); split: 1/60. The oil constituents were expressed as relative proportions without using correcting factors. RIs were calculated using the retention times of a series of *n*‐alkanes (“Target Compounds” software from Perkin‐Elmer).

### 4.4 GC/MS Analysis

The analyses of essential oils were performed with a Perkin‐Elmer TurboMass detector (quadrupole), directly coupled to a Perkin‐Elmer Autosystem XL, equipped with a fused‐silica capillary column (50m × 0.22mm i.d., film thickness 0.25 µm), BP‐1 (dimethylpolysiloxane). Carrier gas, helium at 0.8 mL/min; split, 1/60; injection volume, 0.5 µL; injector temperature, 250°C; oven temperature programmed from 60°C to 220°C at 2°C/min and then held isothermal (20 min); ion source temperature, 250°C; energy ionization, 70 eV; electron ionization mass spectra were acquired over the mass range 40–400 Da.

### 4.5 ^13^C‐NMR Analysis

^13^C‐NMR analysis was acquired on a Bruker AVANCE 400 Fourier Transform spectrometer operating at 100.623 MHz for ^13^C, equipped with a 5 mm probe, in deuterated chloroform (CDCl_3_), using tetramethylsilane (TMS) as internal reference. ^13^C‐NMR spectra were registered with these parameters: pulse width (PW), 4 µs (flip angle 45°); acquisition time, 2.73 s for a 128 K data table with a spectral width (SW) of 220 000 Hz (220 ppm); CPD mode decoupling; digital resolution 0.183 Hz/pt. The number of accumulated scans ranged from 2000 to 3000 for each sample (around 40 mg of oil in 0.5 mL of CDCl_3_). The Fourier transformation was preceded by an exponential line broadening multiplication (1.0 Hz) of the free induction decay.

### 4.6 Identification of Components

Identification of the components was carried out: (a) by comparing of their GC RIs on polar and nonpolar columns, calculated by the retention times of a series of *n*‐alkanes (Target Compounds software of Perkin‐Elmer), with those of authentic compounds and with reference data [30, 31] on computer comparison with commercial mass spectral libraries [32, 33, 34] and (b) by comparing the signals in the ^13^C‐NMR spectra of essential oils with those of reference spectra compiled in the laboratory's spectral library, using in‐house developed software [35]. In the analyzed samples, individual components were identified by NMR at concentrations as low as 0.4%–0.5%.

### 4.7 Data Analysis

PCA and Hierarchical clustering (Ward's method) were performed by XLStat (Adinsoft, France) [36].

## Author Contributions

**Stéphane Gervais Gooré**: sampling and extraction, and original draft preparation. **Bi Tra Marc Gabin Djié**: sampling and extraction. **Bi Goblé Landry Guessan**: sampling and extraction. **Zana Adama Ouattara**: original draft preparation and writing – review. **Thomas Maroselli**: investigation, formal analysis. **Yves‐Alain Békro**: conceptualization, methodology, investigation, formal analysis, original draft preparation, and writing – review. **Mathieu Paoli**: investigation, formal analysis. **Félix Tomi**: conceptualization, methodology, original draft preparation, and writing – review.

## Conflicts of Interest

The authors declare no conflicts of interest.

## Supporting information




**Supporting File**: cbdv71670‐sup‐0001‐S1.docx.

## Data Availability

The data that support the findings of this study are available from the corresponding author upon reasonable request.

## References

[1] WFO plant list, accessed June, 2026, https://wfoplantlist.org/taxon/wfo‐0000397052‐2025‐12?matched_id=wfo‐0000636743.

[2] L. Swana , B. Tsakem , J. V. Tembu , et al., “The Genus *Dacryodes* Vahl.: Ethnobotany, Phytochemistry and Biological Activities‐Review,” Pharmaceuticals 16 (2023): 775, 10.3390/ph16050775. 37242558 PMC10223253

[3] L. H. Tee , B. Yang , B. T. Tey , et al., “Valorization of *Dacryodes rostrata* Fruit Through the Characterization of its Oil,” Food Chemistry 235 (2017): 257–264, 10.1016/j.foodchem.2017.05.021. 28554634

[4] J. M. Onana , “A Synoptic Revision of *Dacryodes* (Burseraceae) in Africa, With a New Species From Central Africa,” Kew Bulletin 63 (2008): 385–400, 10.1007/s12225-008-9064-4.

[5] J. O. Igoli , O. G. Ogaji , T. A. Tor‐Ayiin , and N. P. Igoli , “Traditional Medicine Practice Amongst the Igede People of Nigeria. Part II,” African Journal of Traditional, Complementary and Alternative Medicines 2 (2005): 134–152, 10.4314/ajtcam.v2i2.31112.

[6] E. Adjanohoun and L. A. Assi , “Contribution au Recensement Des Plantes Médicinales De Côte D'Ivoire. Abidjan, Côte d'Ivoire,” in Ministère De la Recherche Scientifique (Centre National de Floristique, 1979), 359.

[7] K. K. Ajibesin , B. A. Ekpo , D. N. Bala , E. E. Essien , and S. A. Adesanya , “Ethnobotanical Survey of Akwa Ibom State of Nigeria,” Journal of Ethnopharmacology 115 (2008): 387–408, 10.1016/j.jep.2007.10.021. 18053664

[8] Useful Tropical Plants Home Page, accessed June, 2026, https://tropical.theferns.info/viewtropical.php?id=Dacryodes+klainea–na.

[9] P. E. Obi , K. O. Ebuoh‐Ike , and C. E. Obi , “Anti‐Diabetic Activity of Ethanol Stem Bark Extract of *Dacryodes klaineana* (Pierre) H. J. Lam (Burseraceae) on Alloxan Induced Diabetic Rats,” Trends in Natural Products Research 5 (2024): 72–80.

[10] A. L. Oseigbokhan , R. B. Olabisi , L. I. Oduola , and O. R. Ogheneovo , “Phytochemical Screening and Mineral Potentials of the Kernel of *Dacryodes klaineana* (Pierre) H.J. Lam,” Open Science Journal of Analytical Chemistry 4 (2019): 7–12.

[11] O. R. Ogheneovo , A. L. Oseigbokan , R. B. Olabisi , and L. I. Oduola , “Phytochemical Screening and Mineral Analysis of the Pulp of *Dacryodes klaineana* (Pierre) H.J. LAM,” American Journal of Chemical and Materials Science 6 (2019): 21–24.

[12] L. Guang‐Yi , C. D. Bates , A. I. Gray , and P. G. Waterman , “The Volatile Oil of the Oleo Resin of *Aucoumea klaineana* Collected in Gabon,” Planta Medica 54 (1988): 368–369, 10.1055/s-2006-962467. 17265296

[13] J. Koudou , L.‐C. Obame , B. S. Kumulungui , et al., “Volatile Constituents and Antioxidant Activity of *Aucoumea klaineana* Pierre Essential Oil,” African Journal of Pharmacy and Pharmacology 3 (2009): 323–326.

[14] P. M. J. Dongmo , F. Tchoumbougnang , B. Ndongson , et al., “Chemical Characterization, Antiradical, Antioxidant and Anti‐Inflammatory Potential of the Essential Oils of *Canarium schweinfurthii* and *Aucoumea klaineana* (Burseraceae) Growing in Cameroon,” Agriculture and Biology Journal of North America 1 (2010): 606–611.

[15] J. B. Boti , G. Koukoua , T. Y. N'Guessan , and J. Casanova , “Chemical Variability of *Conyza sumatrensis* and *Microglossa pyrifolia* From Côte d'Ivoire,” Flavour and Fragrance Journal 22 (2007): 27–31, 10.1002/ffj.1743.

[16] T. A. Yapi , J. B. Boti , C. A. Ahibo , et al., “Chemical Variability of the Leaf Essential Oil of *Xylopia aethiopica* (Dunal) A.Rich. From Côte d'Ivoire,” Chemistry & Biodiversity 9 (2012): 2802–2809, 10.1002/cbdv.201200145. 23255449

[17] Z. A. Ouattara , J. B. Boti , K. B. Attioua , et al., “Chemical Variability of *Cleistopholis patens* (Benth.) Engl. Et Diels Leaf Oil From Ivory Coast,” Chemistry & Biodiversity 10 (2013): 2053–2060, 10.1002/cbdv.201300076. 24243614

[18] S. G. Gooré , Z. A. Ouattara , T. A. Yapi , et al., “Chemical Composition of Ivorian *Artabotrys insignis* Leaf Oil. Combined Analysis Including 13C NMR, to Quantify Germacrene A and β‐Elemene,” Natural Product Research 31, no. 15 (2017): 1836–1839, 10.1080/14786419.2017.1292269. 28278653

[19] D. A. Kambiré , J. B. Boti , A. C. L. Kablan , et al., “Chemical Variability and In Vitro Anti‐Inflammatory Activity of Leaf Essential Oil From Ivorian *Isolona dewevrei* (De Wild. & T. Durand) Engl. & Diels,” Molecules 26, no. 20 (2021): 6228, 10.3390/molecules26206228. 34684809 PMC8539547

[20] F. Tomi , P. Bradesi , A. Bighelli , and J. Casanova , “Computer‐Aided Identification of Individual Components of Essential Oils Using Carbon‐13 NMR Spectroscopy,” Journal of Magnetic Resonance Analysis 1 (1995): 25–34.

[21] S. G. Gooré , Z. A. Ouattara , A. T. Yapi , et al., “Chemical Composition of the Leaf Oil of *Artabotrys jollyanus* From Côte d'Ivoire,” Revista Brasileira de Farmacognosia 27 (2017): 414–418, 10.1016/j.bjp.2017.04.001.

[22] M. D. Eggers , V. Sinnwell , and E. Stahl‐Biskup , “(–)‐Spirolepechinene, a Spirosesquiterpene From *Lepechinia bullata* (Lamiaceae),” Phytochemistry 51 (1999): 987–990, 10.1016/S0031-9422(99)00048-5.

[23] T. Zhao , Caractérisations Chimiques et Biologiques D'extraits De Plantes Aromatiques et Médicinales Oubliées Ou Sous‐utilisées de Midi‐Pyrénées (France) et Chongqing (Chine) (PhD diss., Institut National Polytechnique de Toulouse– Université de Toulouse, 2014): 174.

[24] J. Koudou , P. Edou , L. C. Obame , et al., “Volatile Components, Antioxidant and Antimicrobial Properties of the Essential Oil of *Dacryodes edulis* G. Don From Gabon,” Journal of Applied Sciences 8 (2008): 3532–3535, 10.3923/jas.2008.3532.3535.

[25] E. Valarezo , S. Ojeda‐Riascos , L. Cartuche , N. Andrade‐González , I. González‐Sánchez , and M. A. Meneses , “Extraction and Study of the Essential Oil of Copal (*Dacryodes peruviana*), an Amazonian Fruit With the Highest Yield Worldwide,” Plants 9 (2020): 1658, 10.3390/plants9121658. 33256174 PMC7760007

[26] L. C. Espinoza , E. Valarezo , M. J. Fábrega , et al., “Characterization and In Vivo Anti‐Inflammatory Efficacy of Copal (*Dacryodes peruviana* (Loes.) H.J. Lam) Essential Oil,” Plants 11 (2022): 3104, 10.3390/plants11223104. 36432834 PMC9696342

[27] S. H. Riwom , F. M.‐C. N. Foe , M. A. Nyegue , R. A. Wanki , S. V. Olugu , and F.‐X. Etoa , “Chemical Composition and In Vitro Antibacterial Activity of the Essential Oils of the Leaves, Resin and Stem‐Barks of *Dacryodes edulis* (G. Don) H. J Lam Growing in Cameroon on Diarrhea Associated Strains,” Journal of Applied Pharmaceutical Science 5 (2015): 006–011, 10.7324/JAPS.2015.501002.

[28] T. Silou , A. N. Loumouamou , S. Goténi , et al., “Variability of Essential Oils From the Leaves of the Safou Tree *Dacryodes edulis* (G. Don) H.J. LAM in Congo‐Brazzaville,” Journal of Essential Oil Bearing Plants 15 (2012): 108–115, 10.1080/0972060X.2012.10644026.

[29] P. A. Onocha , O. Ekundayo , O. Oyelola , and I. Laakso , “Essential Oils of *Dacryodes edulis* (G. Don) H. J. Lam (African pear),” Flavour and Fragrance Journal 14 (1999): 135–139, 10.1002/(SICI)1099-1026(199903/04)14:2<135::AID-FFJ788>3.0.CO;2-L.

[30] Z. A. Ouattara , J. B. Boti , A. C. Ahibo , et al., “The Key Role of ^13^C NMR Analysis in the Identification of Individual Components of *Polyalthia longifolia* Leaf Oil,” Flavour and Fragrance Journal 29 (2014): 371–379, 10.1002/ffj.3215.

[31] S. G. Gooré , Z. A. Ouattara , T. A. Yapi , et al., “Chemical Composition of Ivorian *Artabotrys insignis* Leaf Oil. Combined Analysis Including ^13^C NMR, to Quantify Germacrene A and β‐Elemene,” Natural Product Research 31 (2017): 1836–1839, 10.1080/14786419.2017.1292269. 28278653

[32] W. A. Konig , D. H. Hochmuth , and D. Joulain , Terpenoids and Related Constituents of Essential Oils (Institute of Organic Chemistry, 2001), Library of Mass Finder 2.1.

[33] R. P. Adams , Identification of Essential Oils Components by Gas Chromatography/Mass Spectroscopy (Allured, 2007).

[34] , PC Version of the Mass Spectral Library (National Institute of Standards and Technology, 2014).

[35] O. Bazzali , T. H. Thai , T. M. Hoi , et al., “Integrated Analysis of the Wood Oil From *Xanthocyparis vietnamensis* Farjon & Hiep. By Chromatographic and Spectroscopic Techniques,” Molecules 21 (2016): 840, 10.3390/molecules21070840. 27355937 PMC6273169

[36] P. Legendre and L. Legendre , Numerical Ecology (Elsevier Science BV, 1998).

