# Supplementary material for: Composition and Chemical Variability of Trunk Bark Oil of Pachylobus klaineanus (Pierre) Guillaumin. From Côte D'Ivoire
Source: Chem Biodivers. 2026 Sep 3;23(9):e71670. doi: 10.1002/cbdv.71670 (PMC13539467; doi:10.1002/cbdv.71670)
Supplement: Supplementary file 1 [file CBDV-23-e71670-s001.docx]

**SUPPLEMENTARY MATERIALS**

Table S1. Chemical composition of trunk bark essential oil from *P klaineanus*.

|  | **Components** | **Adiopodoumé** | | | | | | | | | | | | | | **Dzeudji** | | | | | |
| --- | --- | --- | --- | --- | --- | --- | --- | --- | --- | --- | --- | --- | --- | --- | --- | --- | --- | --- | --- | --- | --- |
|  |  | **Ad1** | **Ad2** | **Ad3** | **Ad4** | **Ad5** | **Ad6** | **Ad7** | **Ad8** | **Ad9** | **Ad10** | **Ad11** | **Ad12** | **Ad13** | **Ad14** | **Dz15** | **Dz16** | **Dz17** | **Dz18** | **Dz19** | **Dz20** |
| 1 | α-Thujene | 0.5 | 0.9 | 4.5 | 0.8 | 2.8 | 1.9 | 1.3 | 1.5 | 0.5 | 0.9 | 1.7 | 0.8 | 1.6 | 0.5 | 3.8 | 2.9 | 3.9 | 1.6 | 1.4 | 1.6 |
| 2 | α-Pinene | 36.3 | 38.8 | 46.4 | 44.6 | 42.2 | 74.5 | 45.3 | 56.2 | 50.3 | 66.6 | 22.6 | 55.7 | 32.9 | 55.6 | 43.1 | 41.5 | 60.9 | 41.2 | 46.3 | 49.0 |
| 3 | α-Fenchene | tr | 0.1 | tr | tr | 0.1 | tr | 0.1 | tr | tr | tr | 0.1 | tr | 0.1 | 0.1 | 0.1 | 0.1 | tr | 0.1 | 0.1 | 0.1 |
| 4 | Camphene | 0.2 | 0.6 | 2.7 | 0.8 | 0.8 | 0.5 | 0.8 | 0.4 | 0.5 | 1.1 | 1.0 | 0.3 | 1.0 | 1.0 | 0.5 | 0.3 | 0.4 | 1.2 | 2.1 | 0.7 |
| 5 | Thuja-2,4(10)-diene | 0.1 | - | - | - | - | - | - | - | - | - | 0.1 | - | - | - | 0.1 | - | - | - | 0.1 | 0.2 |
| 6 | Sabinene | 0.2 | 0.5 | 6.0 | 0.2 | 2.0 | 0.7 | 0.6 | 0.5 | 0.2 | 0.2 | 1.0 | 0.2 | 0.4 | 0.2 | 3.6 | 1.2 | 1.7 | 0.3 | 0.7 | 1.4 |
| 7 | β-Pinene | 5.5 | 22.3 | 16.6 | 35.1 | 34.2 | 10.2 | 25.3 | 16.3 | 16.5 | 11.4 | 30.2 | 6.1 | 46.3 | 28.1 | 8.4 | 7.4 | 10.0 | 32.1 | 13.7 | 4.8 |
| 8 | Myrcene | 17.9 | 1.1 | 2.0 | 0.3 | 3.5 | 0.5 | 0.4 | 0.9 | 0.5 | 0.2 | 0.9 | 0.5 | 0.1 | 0.4 | 0.7 | 1.5 | 6.2 | 0.2 | 0.6 | 0.2 |
| 9 | α-Phellandrene | - | 0.1 | 0.3 | 0.1 | 0.1 | 0.1 | 0.1 | 0.2 | 0.1 | 0.1 | 0.2 | 0.2 | 0.1 | 0.2 | 0.2 | 0.2 | 0.1 | 0.1 | - | tr |
| 10 | δ-3-Carene | 0.1 | - | - | - | - | 0.1 | - | - | - | - | - | - | - | - | - | 0.1 | - | tr | - | 0.1 |
| 11 | α-Terpinene | - | 0.4 | 1.8 | 0.2 | 0.8 | 0.4 | 0.4 | 0.5 | 0.2 | 0.2 | 0.8 | 0.3 | 0.3 | 0.2 | 2.5 | 0.6 | 0.8 | 1.2 | - | 0.2 |
| 12 | p-Cymene | 1.0 | 0.5 | 2.4 | 0.4 | 1.0 | 0.5 | 0.8 | 0.5 | 0.2 | 0.2 | 1.5 | 0.3 | 0.3 | 0.1 | 6.0 | 3.2 | 1.5 | 3.9 | 3.7 | 3.2 |
| 13 | Limonene | 0.9 | 2.7 | 1.4 | 2.8 | 1.5 | 1.2 | 1.8 | 2.6 | 1.8 | 2.3 | 6.6 | 1.4 | 2.5 | 2.1 | 1.9 | 5.5 | 1.1 | 3.3 | 5.9 | 1.5 |
| 14 | β-Phellandrene | 0.6 | 1.3 | 1.5 | 1.4 | 1.2 | 0.9 | 1.1 | 1.6 | 1.5 | 1.3 | 0.9 | 1.0 | 1.8 | 1.4 | 1.3 | 0.7 | 1.4 | 1.3 | - | 0.5 |
| 15 | (*Z*)-β-Ocimene | 0.4 | 0.1 | tr | tr | - | 0.1 | 0.4 | tr | 0.1 | tr | - | 0.3 | tr | 0.1 | tr | tr | 0.2 | - | - | - |
| 16 | (*E*)-β-Ocimene | 2.9 | 1.5 | 0.1 | 0.1 | 0.1 | 1.3 | 8.2 | 0.3 | 0.1 | 0.1 | 0.1 | 0.2 | tr | tr | 0.1 | tr | 0.3 | - | - | - |
| 17 | γ-Terpinene | tr | 0.9 | 3.4 | 0.4 | 1.6 | 0.9 | 0.7 | 1.0 | 0.4 | 0.4 | 1.2 | 0.5 | 0.6 | 0.3 | 4.1 | 1.2 | 1.7 | 1.5 | tr | 0.5 |
| 18 | p-Cymenene | 0.1 | 0.1 | tr | tr | tr | tr | - | tr | - | - | 1.4 | - | - | - | 0.3 | 0.8 | 0.1 | 0.1 | 1.9 | 0.4 |
| 19 | Terpinolene | 0.1 | 0.7 | 0.9 | 0.4 | 0.6 | 0.4 | 0.6 | 0.7 | 0.4 | 0.3 | 19.2 | 0.4 | 0.6 | 0.5 | 3.3 | 15.0 | 0.7 | 0.7 | tr | 0.4 |
| 20 | Linalool | - | 0.1 | 0.1 | - | - | - | - | - | - | - | - | - | - | - | 0.1 | 0.1 | - | - | - | 0.1 |
| 21 | Fenchol | - | 0.1 | - | 0.1 | 0.1 | - | 0.1 | tr | - | - | 0.1 | tr | 0.1 | 0.1 | 0.1 | 0.1 | 0.1 | 0.3 | - | 0.2 |
| 22 | *trans*-Pinocarveol | 0.3 | 0.2 | 0.2 | 0.1 | 0.1 | - | 0.1 | - | - | - | 0.4 | - | - | - | 0.2 | 0.2 | 0.1 | 0.1 | 0.9 | 0.5 |
| 23 | Pinovarvone | - | - | - | - | - | - | - | - | - | - | - | - | - | - | tr | - | - | - | 0.3 | 0.3 |
| 24 | p-Cymen-8-ol | 0.1 | - | - | tr | - | tr | tr | - | - | - | 1.9 | - | - | - | 0.1 | 1.5 | - | 0.1 | 2.0 | 0.3 |
| 25 | Terpinen-4-ol | 0.4 | 1.0 | 4.8 | 0.5 | 1.9 | 0.8 | 0.8 | 0.6 | 0.3 | 0.2 | 1.9 | 0.4 | 0.5 | 0.2 | 6.9 | 3.4 | 1.5 | 1.0 | 1.6 | 4.0 |
| 26 | Myrtenal | 0.3 | 0.2 | 0.1 | 0.1 | 0.1 | 0.1 | 0.1 | 0.1 | - | - | 0.4 | - | - | - | 0.1 | 0.1 | 0.1 | tr | 1.0 | 0.5 |
| 27 | α-Terpineol | 0.5 | 2.3 | 0.7 | 1.2 | 1.2 | 0.9 | 1.1 | 0.6 | 0.7 | 0.2 | 1.7 | 0.8 | 1.7 | 1.4 | 1.5 | 1.5 | 0.6 | 3.6 | 2.8 | 4.9 |
| 28 | Myrtenol | - | - | - | - | - | - | - | - | - | - | - | - | - | - | - | - | - | - | 0.4 | 0.3 |
| 29 | Verbenone | - | - | - | - | - | - | - | - | - | - | - | - | - | - | - | - | - | - | 0.6 | 0.6 |
| 30 | *trans*-Carveol | - | - | - | - | - | - | - | - | - | - | - | - | - | - | - | - | - | - | 0.3 | 0.3 |
| 31 | Thymol | - | - | - | - | - | - | tr | 0.1 | - | - | - | - | tr | - | - | - | - | - | - | - |
| 32 | α-Cubebene | 0.4 | 0.1 | 0.1 | 0.2 | - | tr | 0.1 | 0.2 | 0.1 | 0.1 | - | 0.1 | tr | tr | 0.1 | 0.1 | 0.1 | - | - | 0.5 |
| 33 | Cyclosativene | 0.2 | - | - | 0.2 | - | - | 0.1 | - | 0.1 | - | - | tr | - | - | 0.2 | - | - | - | - | 0.9 |
| 34 | α-Ylangene | - | 0.2 | - | - | - | - | - | - | - | 0.2 | - | - | - | - | - | - | - | - | - | 0.2 |
| 35 | α-Copaene | 1.2 | 2.1 | 1.1 | 0.6 | 0.4 | 0.1 | 0.4 | 0.4 | 0.6 | 0.2 | tr | 0.2 | 0.1 | 0.1 | 0.6 | 0.3 | 0.4 | 0.1 | 0.1 | 1.6 |
| 36 | β-Cubebene | - | - | - | - | - | - | - | - | - | - | - | - | - | - | - | - | - | - | - | - |
| 37 | β-Elemene | 0.4 | 0.5 | tr | 0.1 | 0.1 | - | 0.2 | 0.5 | 0.3 | 0.2 | - | 0.1 | 0.1 | tr | 0.2 | 0.1 | 0.1 | 0.1 | - | - |
| 38 | Cyperene | 0.2 | 0.1 | tr | 0.1 | 0.6 | - | 0.1 | 0.9 | - | - | - | tr | 0.2 | tr | - | 0.2 | 0.8 | 0.7 | 0.4 | 0.1 |
| 39 | α-Gurjunene | 3.1 | - | - | - | - | 0.1 | 0.3 | 0.2 | - | - | 1.2 | - | - | - | - | - | - | - | - | - |
| 40 | *cis*-α-Bergamotene | - | 1.1 | - | - | - | - | - | - | 0.2 | - | - | - | - | - | - | - | - | - | - | - |
| 41 | α-Santalene | 0.1 | - | - | - | - | - | - | - | - | 0.1 | - | - | - | - | 0.2 | 0.1 | - | - | - | - |
| 42 | (*E*)-β-Caryophyllene | 0.7 | 1.0 | 0.1 | 0.6 | 0.1 | 0.5 | 0.3 | 0.6 | 0.7 | 0.2 | - | 3.1 | 0.5 | 0.5 | 0.3 | 0.6 | 0.2 | 0.2 | 0.2 | - |
| 43 | β-Copaene | 0.1 | - | - | 0.1 | - | - | - | - | - | - | - | - | tr | - | 1.0 | - | - | tr | - | 0.4 |
| 44 | γ-Elemene | - | - | - | - | - | - | - | - | - | - | - | - | - | - | - | - | - | - | - | - |
| 45 | *trans*-α-Bergamotene | - | - | - | - | - | - | - | - | - | - | - | - | - | - | - | - | - | - | - | - |
| 46 | α-Guaiene | - | - | - | - | - | - | - | - | 0.6 | - | - | - | - | - | - | - | - | - | - | - |
| 47 | Aromadendrene | 1.1 | - | - | - | - | - | - | 0.6 | 0.5 | - | - | - | - | - | - | - | - | - | - | - |
| 48 | Spirolepechinene | - | - | - | - | - | - | - | - | - | - | - | - | - | - | - | - | - | - | - | - |
| 49 | (*E*)-β-Farnesene | - | - | - | - | - | - | - | - | - | - | - | - | - | - | - | - | 0.9 | - | - | - |
| 50 | α-Humulene | 0.8 | 0.4 | tr | 0.8 | - | 0.7 | 0.1 | 0.2 | 0.2 | 0.1 | - | 6.4 | 0.2 | 0.8 | 0.1 | 0.1 | - | - | - | - |
| 51 | Rotundene | - | - | - | - | - | - | - | 0.6 | - | 0.1 | - | 0.2 | 0.1 | 0.1 | - | - | 0.6 | 0.5 | - | - |
| 52 | *allo*-Aromadendrene | 1.7 | 0.6 | tr | 0.4 | - | - | 0.5 | 0.6 | 2.5 | 0.4 | - | 0.9 | 0.3 | 0.3 | 0.6 | 0.1 | - | - | 0.2 | 0.4 |
| 53 | Ishwarane | - | - | - | - | - | - | - | - | - | - | - | - | - | - | - | 0.2 | - | - | 0.1 | 0.1 |
| 54 | γ-Muurolene | 0.1 | 0.1 | tr | 0.2 | - | - | 0.1 | 0.2 | - | 0.5 | - | 0.1 | 0.1 | - | 0.1 | 0.4 | tr | 0.1 | 0.1 | 1.1 |
| 55 | Germacrene D | - | 0.6 | - | - | - | 0.1 | 0.1 | 0.9 | - | 0.5 | - | 0.1 | - | - | - | - | - | - | - | - |
| 56 | γ-Humulene | - | 2.1 | - | - | - | - | - | - | - | 2.2 | - | - | - | - | - | - | tr | - | - | - |
| 57 | β-Selinene | 0.4 | 2.3 | - | 0.1 | - | 0.7 | 0.2 | - | - | 0.4 | - | 1.2 | 1.2 | - | 1.2 | - | 0.1 | 0.1 | 0.1 | 0.6 |
| 58 | α-Selinene | - | 1.6 | - | 0.2 | - | 0.4 | 0.2 | - | 0.5 | 0.3 | - | 1.1 | 0.8 | 0.1 | 0.7 | - | 0.1 | - | - | - |
| 59 | Isodaucene | - | - | - | - | - | - | - | - | - | - | - | - | - | - | - | - | - | - | 2.2 | - |
| 60 | α-Muurolene | 0.2 | - | - | - | - | - | - | - | - | - | - | - | - | - | 0.3 | 0.3 | - | - | - | - |
| 61 | (*E*),(*E*)-α-Farnesene | - | - | - | - | - | - | - | - | - | - | - | - | - | - | - | - | - | - | - | - |
| 62 | α-Bulnesene | - | - | - | - | - | - | - | - | 0.7 | - | - | - | - | - | - | - | - | - | - | - |
| 63 | β-Bisabolene | - | - | - | - | - | - | - | - | - | - | - | - | - | - | - | - | - | - | - | - |
| 64 | γ-Cadinene | 2.6 | 1.7 | - | 0.6 | - | - | 1.6 | 0.3 | 1.6 | 1.8 | - | 3.0 | 1.0 | 1.1 | 0.7 | 0.6 | - | 1.6 | - | 2.3 |
| 65 | *trans*-Calamenene | 1.4 | - | - | 0.2 | - | - | 0.2 | 0.4 | 0.3 | 0.2 | - | 0.2 | 0.1 | - | - | - | - | - | - | 0.4 |
| 66 | δ-Cadinene | 0.7 | 1.4 | 0.5 | 0.6 | 0.3 | 0.1 | 0.4 | 0.5 | 0.7 | 0.9 | - | 0.9 | 0.4 | 0.3 | 0.3 | 0.7 | 0.2 | - | - | 1.2 |
| 67 | α-Calacorene | - | - | - | - | - | - | - | - | - | - | - | - | - | - | - | - | - | - | - | - |
| 68 | Selina-4(15),7(11)-diene | - | - | - | - | - | - | - | - | - | - | - | - | - | - | - | - | - | - | - | - |
| 69 | β-Elemol | - | - | - | - | - | - | - | - | - | - | - | - | - | - | - | - | - | - | - | - |
| 70 | Selina-3,7(11)-diene | - | - | - | - | - | - | - | - | - | - | - | - | - | - | - | - | - | - | - | - |
| 71 | (*E*)-Nerolidol | 0.8 | - | - | 0.4 | - | - | - | - | - | - | - | - | - | 0.1 | - | 0.1 | tr | - | - | - |
| 72 | Germacrène B | - | - | - | - | - | - | - | - | - | - | - | - | - | - | - | - | - | - | - | - |
| 73 | Spathulenol | 0.7 | - | - | - | 0.1 | - | - | 0.2 | - | - | - | - | - | - | - | tr | - | - | 0.1 | 0.1 |
| 74 | Caryophyllene oxide | 0.3 | 0.1 | - | 0.1 | - | tr | - | - | 0.1 | - | - | 0.1 | tr | - | tr | 0.1 | - | - | 0.1 | 0.1 |
| 75 | Viridiflorol | - | - | - | - | - | - | - | - | - | - | - | - | - | - | - | - | - | - | - | - |
| 76 | Ledol | 0.5 | - | - | - | - | - | - | - | - | - | - | - | - | - | - | - | - | - | - | - |
| 77 | Humulene oxide II | 0.3 | - | - | - | - | - | - | - | - | - | - | - | - | 0.1 | - | - | - | - | - | - |
| 78 | 1,10-di-*epi*-Cubenol | 0.3 | 0.3 | - | 0.2 | - | - | 0.3 | - | 0.2 | 0.2 | - | 0.7 | 0.2 | 0.2 | 0.1 | 0.1 | - | 0.2 | - | 0.3 |
| 79 | τ-Cadinol | 5.1 | 3.9 | tr | 2.1 | - | - | 3.6 | 0.5 | 5.2 | 3.5 | - | 8.9 | 2.4 | 2.9 | 1.5 | 1.0 | tr | - | - | 4.1 |
| 80 | τ-Muurolol | - | - | - | - | - | - | - | - | - | - | - | - | - | - | - | - | - | - | - | 0.3 |
| 81 | β-Eudesmol | 0.5 | - | - | - | - | - | - | - | - | - | - | - | - | - | - | - | - | - | - | - |
| 82 | α-Cadinol | 0.2 | - | - | - | - | - | - | - | - | - | - | - | - | - | - | - | - | - | - | 0.5 |
| 83 | α-Eudesmol | 0.3 | - | - | - | - | - | - | - | - | - | - | - | - | - | - | - | - | - | - | - |
|  |  |  |  |  |  |  |  |  |  |  |  |  |  |  |  |  |  |  |  |  |  |
|  |  | **92.7** | **96.4** | **97.9** | **97.5** | **97.3** | **98.7** | **98.6** | **92.4** | **89.6** | **97.9** | **97.1** | **97.0** | **98.9** | **99.1** | **97.0** | **94.3** | **97.1** | **97.5** | **90.2** | **91.9** |

|  | **Components** | **Dzeudji** | | | | | | | | **Yapo abbé** | | | | | | | | | | | |
| --- | --- | --- | --- | --- | --- | --- | --- | --- | --- | --- | --- | --- | --- | --- | --- | --- | --- | --- | --- | --- | --- |
|  |  | **Dz21** | **Dz22** | **Dz23** | **Dz24** | **Dz25** | **Dz26** | **Dz27** | **Dz28** | **Ya29** | **Ya30** | **Ya31** | **Ya32** | **Ya33** | **Ya34** | **Ya35** | **Ya36** | **Ya37** | **Ya38** | **Ya39** | **Ya40** |
| 1 | α-Thujene | 1.0 | 0.8 | 0.3 | 0.7 | 2.5 | 6.3 | 1.7 | 1.6 | 1.1 | 1.7 | 0.6 | 2.1 | 1.7 | 0.8 | 0.5 | 5.5 | 0.4 | 2.5 | 0.5 | 0.3 |
| 2 | α-Pinene | 32.3 | 51.8 | 16.1 | 43.9 | 22.2 | 44.8 | 60.8 | 35.5 | 42.4 | 41.6 | 51.7 | 40.2 | 67.2 | 63.4 | 27.6 | 41.6 | 41.8 | 54.5 | 67.8 | 60.9 |
| 3 | α-Fenchene | 0.1 | 0.1 | tr | 0.1 | 0.2 | 0.1 | 0.1 | 0.1 | 0.1 | 0.1 | 0.1 | 0.2 | tr | 0.1 | tr | 0.1 | tr | tr | 0.1 | 0.1 |
| 4 | Camphene | 0.8 | 0.3 | 0.3 | 1.1 | 1.2 | 0.4 | 0.4 | 0.7 | 0.9 | 1.1 | 1.1 | 1.1 | 0.2 | 0.4 | 0.3 | 0.9 | 0.2 | 0.2 | 0.3 | 0.4 |
| 5 | Thuja-2,4(10)-diene | 0.2 | 0.2 | 0.1 | 0.3 | 0.1 | 0.2 | 0.3 | 0.1 | - | - | - | - | - | - | - | - | - | 0.2 | 0.5 | - |
| 6 | Sabinene | 0.2 | 0.5 | 0.3 | 0.3 | 1.7 | 3.7 | 0.5 | 0.3 | 0.2 | 1.1 | 0.1 | 0.6 | 0.2 | 0.4 | 0.3 | 1.9 | 0.1 | 1.2 | 0.2 | 0.1 |
| 7 | β-Pinene | 14.8 | 4.9 | 9.4 | 21.8 | 24.6 | 5.7 | 5.6 | 20.1 | 33.8 | 29.5 | 27.6 | 20.2 | 6.9 | 8.0 | 3.8 | 24.4 | 4.0 | 5.4 | 4.3 | 7.0 |
| 8 | Myrcene | 1.3 | 0.6 | 0.2 | 1.1 | 0.1 | 0.5 | 0.2 | 1.9 | 7.6 | 3.7 | 0.6 | 0.5 | 0.4 | 0.8 | 0.7 | 0.4 | 0.1 | 0.2 | 0.1 | 0.6 |
| 9 | α-Phellandrene | 0.1 | - | - | - | - | - | 0.1 | 0.2 | tr | 0.1 | 0.1 | 0.2 | 0.1 | 0.1 | 19.8 | 0.1 | tr | 0.1 | - | 0.1 |
| 10 | δ-3-Carene | 0.1 | - | - | - | - | - | - | - | tr | - | tr | - | 0.1 | - | tr | - | - | 0.1 | - | tr |
| 11 | α-Terpinene | 0.2 | - | - | - | - | 0.1 | 0.2 | 0.3 | 0.1 | 0.6 | 0.1 | 1.3 | 0.3 | 0.3 | 0.3 | 0.7 | 0.1 | 0.4 | - | 0.1 |
| 12 | p-Cymene | 2.0 | 1.6 | 2.1 | 1.7 | 8.1 | 11.7 | 4.2 | 0.9 | 1.0 | 1.0 | 0.4 | 1.2 | 0.7 | 0.8 | 12.7 | 3.1 | 0.4 | 2.3 | 1.6 | 0.5 |
| 13 | Limonene | 13.8 | 11.2 | 8.8 | 11.9 | 10.0 | 1.6 | 1.6 | 13.8 | 1.7 | 2.3 | 3.3 | 3.0 | 1.8 | 3.6 | 3.5 | 1.8 | 0.9 | 7.0 | 2.5 | 7.5 |
| 14 | β-Phellandrene | 0.4 | 0.3 | 0.2 | 0.3 | 0.7 | 0.8 | 0.6 | 0.6 | 1.0 | 1.4 | 1.1 | 1.2 | 0.8 | 0.8 | 4.9 | 1.3 | 0.4 | 0.4 | 0.2 | - |
| 15 | (*Z*)-β-Ocimene | - | - | - | - | - | - | - | - | 0.1 | 0.1 | - | - | - | - | tr | tr | - | - | - | 0.3 |
| 16 | (*E*)-β-Ocimene | - | - | - | - | - | - | - | tr | 1.2 | 0.8 | tr | - | - | 0.1 | - | 0.3 | - | - | - | 1.2 |
| 17 | γ-Terpinene | 0.4 | 0.2 | - | 0.2 | 0.3 | 0.7 | 0.5 | 0.4 | 0.3 | 1.3 | 0.1 | 2.3 | 0.6 | 0.4 | 0.4 | 1.8 | 0.2 | 1.0 | tr | 0.2 |
| 18 | p-Cymenene | 2.1 | 0.5 | 0.8 | 0.3 | 0.6 | 0.4 | 0.6 | 1.0 | - | tr | 0.1 | 0.1 | 0.2 | 0.3 | 0.1 | 0.2 | 0.1 | 0.3 | 1.8 | tr |
| 19 | Terpinolene | 5.9 | 0.5 | - | 0.2 | 0.3 | 0.3 | 0.5 | 11.4 | 0.3 | 0.9 | 4.6 | 1.6 | 3.8 | 9.8 | 0.6 | 0.8 | 0.3 | 0.4 | 0.3 | 0.6 |
| 20 | Linalool | - | 0.1 | - | 0.2 | - | - | 0.1 | - | - | - | - | - | 0.1 | - | - | - | - | - | - | - |
| 21 | Fenchol | 0.2 | - | - | - | 0.4 | - | 0.2 | 0.1 | 0.1 | - | - | - | - | - | - | - | tr | - | - | - |
| 22 | *trans*-Pinocarveol | 0.8 | 0.5 | 0.8 | 0.7 | 1.2 | 0.6 | 0.7 | 0.2 | 0.1 | - | - | - | - | - | - | - | - | 0.4 | 0.7 | - |
| 23 | Pinovarvone | 0.6 | - | 1.5 | 0.8 | 1.1 | - | 0.7 | - | - | - | - | - | - | - | - | - | - | 0.4 | 0.6 | - |
| 24 | p-Cymen-8-ol | 1.8 | 0.4 | 0.4 | 0.2 | 0.3 | 0.4 | 0.3 | 1.0 | tr | tr | 0.4 | tr | 0.2 | 0.6 | - | 0.1 | tr | 0.2 | 0.9 | tr |
| 25 | Terpinen-4-ol | 1.6 | 1.3 | 1.2 | 0.8 | 4.7 | 7.9 | 3.3 | 0.8 | 0.5 | 1.4 | 0.3 | 3.1 | 0.5 | 0.9 | 0.5 | 4.1 | 0.4 | 2.2 | 0.5 | 0.4 |
| 26 | Myrtenal | 1.0 | 0.7 | 1.6 | 1.2 | 1.7 | 0.6 | 0.8 | 0.3 | 0.1 | 0.1 | 0.1 | tr | tr | 0.1 | - | 0.2 | 0.1 | 0.5 | 0.7 | 0.1 |
| 27 | α-Terpineol | 3.6 | 1.5 | 1.7 | 1.1 | 4.8 | 1.9 | 2.5 | 1.4 | 2.1 | 1.9 | 2.2 | 8.5 | 0.8 | 1.9 | 0.6 | 3.8 | 1.3 | 1.3 | 0.8 | 2.3 |
| 28 | Myrtenol | 0.5 | - | 0.5 | - | 0.9 | 0.2 | - | - | - | - | - | - | - | - | - | - | - | - | - | - |
| 29 | Verbenone | 1.0 | 0.8 | - | - | - | 0.6 | 0.7 | 0.2 | - | - | - | - | - | - | - | - | - | 0.6 | 0.9 | - |
| 30 | *trans*-Carveol | 0.4 | 0.4 | 0.3 | - | - | 0.5 | 0.5 | - | - | - | - | - | - | - | - | - | - | 0.4 | 0.4 | - |
| 31 | Thymol | - | - | - | - | - | - | - | - | - | - | - | 1.3 | - | - | - | - | - | - | - | - |
| 32 | α-Cubebene | - | - | 0.2 | - | - | - | 0.1 | - | tr | - | - | - | 0.1 | - | 0.3 | tr | 0.7 | 0.1 | - | - |
| 33 | Cyclosativene | - | 0.2 | - | - | - | - | - | - | - | - | - | - | 0.2 | - | - | 0.1 | 0.1 | - | - | - |
| 34 | α-Ylangene | - | - | - | - | - | - | - | - | - | - | - | - | - | - | - | - | 0.3 | - | - | - |
| 35 | α-Copaene | 0.1 | 0.4 | 0.8 | 0.3 | 0.3 | - | 0.8 | 0.1 | 0.1 | 0.3 | 0.2 | 0.2 | 0.4 | - | 5.4 | 0.2 | 2.1 | 0.2 | - | 0.4 |
| 36 | β-Cubebene | - | - | - | - | - | - | - | - | - | - | - | - | - | - | - | - | 0.1 | - | - | - |
| 37 | β-Elemene | - | 0.2 | 0.5 | 0.1 | 0.2 | - | 0.2 | - | tr | 0.1 | 0.1 | 0.1 | 0.1 | - | 0.3 | 0.1 | 0.5 | 0.2 | 0.1 | 0.1 |
| 38 | Cyperene | 2.3 | - | - | - | - | 0.5 | - | 1.2 | 1.8 | 0.1 | 0.1 | 0.3 | 0.2 | - | - | tr | 1.4 | 3.0 | 1.7 | - |
| 39 | α-Gurjunene | - | - | - | - | - | - | - | - | - | - | - | - | - | - | - | - | - | 0.5 | 1.6 | - |
| 40 | *cis*-α-Bergamotene | - | - | 25.7 | 0.1 | - | - | - | - | - | - | - | - | - | - | - | - | - | - | - | - |
| 41 | α-Santalene | - | - | 0.5 | - | - | - | - | - | - | 0.1 | - | - | - | - | - | - | 0.5 | - | - | - |
| 42 | (*E*)-β-Caryophyllene | - | 0.3 | 0.4 | 0.3 | 0.3 | - | - | 0.1 | - | 0.1 | 0.1 | 0.1 | 0.2 | 0.3 | 0.5 | 0.5 | 2.9 | 0.4 | 0.4 | 1.2 |
| 43 | β-Copaene | - | - | 0.3 | - | 0.5 | 0.2 | 1.1 | - | - | - | - | - | 0.1 | - | 0.1 | - | 0.9 | 2.1 | - | 0.9 |
| 44 | γ-Elemene | - | - | - | - | - | - | - | - | - | - | - | - | - | - | - | - | - | - | - | - |
| 45 | *trans*-α-Bergamotene | - | - | 1.8 | 1.3 | 0.6 | - | - | - | - | - | - | - | - | - | - | - | - | - | - | - |
| 46 | α-Guaiene | - | - | - | - | - | - | - | - | - | - | - | - | - | - | - | - | - | - | - | - |
| 47 | Aromadendrene | - | - | - | - | - | 0.4 | - | - | - | - | - | - | - | - | - | - | 0.2 | 0.1 | 0.5 | - |
| 48 | Spirolepechinene | - | - | - | - | - | - | - | - | - | - | - | - | - | - | - | - | - | - | - | - |
| 49 | (*E*)-β-Farnesene | - | - | - | - | - | - | 0.9 | - | - | - | - | - | - | - | - | - | - | - | - | - |
| 50 | α-Humulene | - | 0.1 | - | - | - | - | - | - | - | 0.2 | - | 0.1 | - | - | - | 0.1 | 0.7 | - | 0.6 | 2.1 |
| 51 | Rotundene | 1.9 | - | - | - | - | - | - | 0.9 | - | - | - | 0.1 | - | - | - | - | 1.2 | 2.4 | 0.1 | - |
| 52 | *allo*-Aromadendrene | 0.3 | 0.6 | - | 0.4 | - | 0.3 | 0.1 | 0.1 | - | - | - | 0.3 | - | - | - | - | 0.7 | 0.0 | 0.4 | 0.3 |
| 53 | Ishwarane | - | - | - | - | - | - | 0.2 | - | - | - | - | - | - | - | - | - | 0.8 | 0.5 | 0.3 | - |
| 54 | γ-Muurolene | - | - | 1.5 | 0.1 | - | - | 0.3 | - | - | - | - | - | - | - | 0.2 | - | 4.7 | - | - | - |
| 55 | Germacrene D | - | - | - | - | - | - | - | - | - | - | - | - | - | - | - | - | - | - | - | - |
| 56 | γ-Humulene | - | - | - | - | - | - | - | - | - | - | - | - | - | 1.6 | 2.3 | - | - | - | - | - |
| 57 | β-Selinene | - | 3.9 | 1.8 | - | 0.1 | 0.4 | 0.3 | - | 0.2 | - | - | 0.1 | - | - | 0.3 | 1.3 | - | - | 0.5 | 1.4 |
| 58 | α-Selinene | - | 1.7 | - | - | - | 0.2 | 0.2 | - | 0.2 | - | - | 0.1 | 0.2 | - | 0.3 | 0.7 | - | - | 0.3 | 0.8 |
| 59 | Isodaucene | - | - | - | 1.9 | - | - | - | - | - | - | - | - | - | - | - | - | - | - | - | - |
| 60 | α-Muurolene | 0.8 | - | 0.9 | - | - | - | 0.4 | - | - | 0.1 | - | - | - | - | - | - | - | - | - | - |
| 61 | (*E*),(*E*)-α-Farnesene | - | - | - | - | - | - | - | - | - | - | - | - | - | - | - | - | - | - | - | - |
| 62 | α-Bulnesene | - | - | - | - | - | - | - | - | - | - | - | - | - | - | - | - | - | - | - | - |
| 63 | β-Bisabolene | - | - | 1.7 | - | - | - | - | - | - | - | - | - | - | - | - | - | - | - | - | - |
| 64 | γ-Cadinene | 0.2 | 2.4 | - | - | 0.1 | - | 0.3 | 0.1 | - | 2.5 | - | - | 2.3 | - | 0.2 | - | 3.9 | - | 0.1 | 1.0 |
| 65 | *trans*-Calamenene | - | 0.2 | 0.6 | - | - | - | - | 0.1 | - | - | - | - | - | - | - | - | 1.8 | - | - | - |
| 66 | δ-Cadinene | 0.2 | 0.4 | 0.8 | - | 0.2 | - | 0.3 | 0.2 | - | 0.3 | - | 0.3 | 0.6 | - | 3.1 | 0.1 | 5.9 | - | 0.2 | 0.6 |
| 67 | α-Calacorene | - | - | - | - | - | - | - | - | - | - | - | - | - | - | - | - | 0.9 | - | - | - |
| 68 | Selina-4(15),7(11)-diene | - | - | - | - | - | - | - | - | - | - | - | - | - | - | - | - | - | - | - | - |
| 69 | β-Elemol | - | - | - | - | - | - | - | - | - | - | - | - | - | - | - | - | - | - | - | - |
| 70 | Selina-3,7(11)-diene | - | - | - | - | - | - | - | - | - | - | - | - | - | - | - | - | - | - | - | - |
| 71 | (*E*)-Nerolidol | - | - | - | - | 0.1 | - | - | - | - | - | - | - | - | - | - | - | - | - | - | - |
| 72 | Germacrène B | - | - | - | - | - | - | - | - | - | - | - | - | - | - | - | - | - | - | - | - |
| 73 | Spathulenol | 0.1 | - | - | - | - | 0.2 | - | 0.1 | - | - | - | - | - | - | - | - | - | - | - | - |
| 74 | Caryophyllene oxide | 0.1 | 0.1 | 0.3 | - | 0.1 | 0.1 | 0.1 | - | - | - | - | - | - | - | 0.1 | 0.1 | 0.4 | 0.1 | 0.2 | 0.2 |
| 75 | Viridiflorol | - | - | - | - | - | - | - | - | - | - | - | - | - | - | 0.2 | - | - | 0.2 | - | - |
| 76 | Ledol | - | - | - | - | - | - | - | - | - | - | - | - | - | - | - | - | - | - | 0.1 | - |
| 77 | Humulene oxide II | - | - | - | - | - | - | - | - | - | - | - | - | - | - | - | - | - | - | 0.4 | - |
| 78 | 1,10-di-*epi*-Cubenol | - | 0.3 | - | - | - | - | - | - | - | 0.3 | - | 0.4 | 0.4 | - | - | - | 0.3 | - | - | 0.2 |
| 79 | τ-Cadinol | 0.4 | 3.9 | 0.3 | - | 0.2 | - | 0.3 | 0.3 | - | 2.2 | - | 5.8 | 5.5 | tr | - | tr | 4.9 | 0.2 | - | 3.5 |
| 80 | τ-Muurolol | - | - | 0.2 | - | 0.1 | - | 0.1 | - | - | - | - | - | - | - | - | - | 0.6 | - | - | 0.2 |
| 81 | β-Eudesmol | - | - | - | - | - | - | - | - | - | - | - | - | - | - | - | - | - | - | - | - |
| 82 | α-Cadinol | - | 0.1 | 0.4 | - | 0.2 | - | - | - | - | - | - | - | - | - | - | - | - | - | - | - |
| 83 | α-Eudesmol | - | - | - | - | - | - | - | - | - | - | - | - | - | - | - | - | - | - | - | - |
|  |  |  |  |  |  |  |  |  |  |  |  |  |  |  |  |  |  |  |  |  |  |
|  |  | **93.4** | **94.0** | **85.2** | **93.4** | **90.4** | **92.1** | **93.2** | **96.1** | **97.4** | **97.0** | **94.9** | **96.5** | **96.9** | **95.4** | **90.1** | **96.5** | **87.4** | **92.1** | **93.1** | **95.4** |

|  | **Components** | **Yapo abbé** | | | | **Una** | **Mo** | **Kd** | **Ko** | **Petit yapo** | |
| --- | --- | --- | --- | --- | --- | --- | --- | --- | --- | --- | --- |
|  |  | **Ya41** | **Ya42** | **Ya43** | **Ya44** | **Un45** | **Mo46** | **Kd47** | **Ko48** | **Py49** | **Py50** |
| 1 | α-Thujene | 2.7 | 0.8 | 0.8 | 1.5 | 2.5 | 0.2 | 8.6 | 0.4 | 0.6 | 1.9 |
| 2 | α-Pinene | 48.3 | 67.1 | 76.2 | 36.6 | 42.1 | 41.2 | 3.0 | 27.9 | 50.6 | 28.8 |
| 3 | α-Fenchene | 0.1 | tr | tr | 0.1 | tr | tr | - | tr | 0.1 | 0.1 |
| 4 | Camphene | 0.8 | 0.3 | 0.3 | 0.8 | 0.2 | 0.3 | 0.1 | 0.4 | 1.5 | 1.8 |
| 5 | Thuja-2,4(10)-diene | - | - | - | 0.1 | - | - | - | tr | 0.9 | 0.8 |
| 6 | Sabinene | 1.1 | 0.2 | 0.1 | 0.7 | 1.2 | 0.1 | 9.5 | 0.1 | 0.3 | 1.5 |
| 7 | β-Pinene | 20.8 | 7.2 | 7.8 | 32.8 | 5.8 | 3.9 | 2.0 | 14.7 | 6.4 | 4.8 |
| 8 | Myrcene | 0.1 | 0.2 | 0.3 | 0.5 | 24.6 | 0.7 | 0.9 | 1.6 | tr | 1.1 |
| 9 | α-Phellandrene | tr | 0.1 | 0.1 | 0.1 | 0.7 | - | 0.2 | 8.2 | - | tr |
| 10 | δ-3-Carene | - | tr | - | - | 3.2 | 1.1 | 0.1 | - | - | - |
| 11 | α-Terpinene | 0.2 | 0.3 | 0.2 | 0.6 | 0.9 | - | 0.9 | 0.2 | 0.2 | 0.2 |
| 12 | p-Cymene | 3.0 | 0.4 | 0.3 | 1.2 | 1.5 | 7.6 | 21.4 | 2.3 | 2.2 | 4.8 |
| 13 | Limonene | 1.6 | 6.3 | 1.1 | 2.0 | 0.9 | 1.6 | 8.1 | 11.8 | 4.4 | 1.1 |
| 14 | β-Phellandrene | 0.4 | 0.9 | 0.6 | 1.2 | 1.3 | 0.4 | 1.5 | 3.1 | - | 0.6 |
| 15 | (*Z*)-β-Ocimene | - | tr | - | - | 0.6 | 0.1 | - | - | - | - |
| 16 | (*E*)-β-Ocimene | - | - | tr | 0.1 | 0.1 | 0.2 | - | - | - | - |
| 17 | γ-Terpinene | 0.5 | 0.7 | 0.4 | 1.1 | 1.4 | - | 4.7 | 0.3 | 0.3 | 0.8 |
| 18 | p-Cymenene | 0.3 | 0.1 | 0.1 | 0.1 | tr | 0.2 | 0.7 | tr | 1.1 | 0.7 |
| 19 | Terpinolene | 0.3 | 0.7 | 0.4 | 0.8 | 0.5 | - | 1.5 | 0.5 | 0.2 | 0.3 |
| 20 | Linalool | 0.1 | - | - | - | - | - | 0.1 | - | - | - |
| 21 | Fenchol | 0.1 | 0.1 | - | - | - | - | - | 0.1 | - | - |
| 22 | *trans*-Pinocarveol | 1.0 | 0.1 | - | 0.4 | 0.1 | 0.1 | - | - | 1.1 | 2.1 |
| 23 | Pinovarvone | - | - | - | 0.4 | - | - | - | - | 0.7 | 1.4 |
| 24 | p-Cymen-8-ol | 0.3 | - | - | 0.1 | tr | 0.1 | - | - | 0.2 | 0.6 |
| 25 | Terpinen-4-ol | 1.8 | 0.8 | 0.3 | 1.7 | 1.7 | 0.1 | 16.1 | 0.3 | 0.4 | 4.0 |
| 26 | Myrtenal | 1.1 | 0.2 | tr | 0.4 | tr | 0.1 | - | - | 1.4 | 2.1 |
| 27 | α-Terpineol | 1.3 | 1.8 | 1.1 | 3.0 | 0.7 | 0.9 | 1.0 | 1.0 | 0.4 | 1.0 |
| 28 | Myrtenol | - | - | - | - | - | - | - | - | 0.4 | 1.1 |
| 29 | Verbenone | - | - | - | 0.2 | - | - | - | - | 1.0 | 2.9 |
| 30 | *trans*-Carveol | - | - | - | 0.1 | - | - | - | - | 0.4 | 0.9 |
| 31 | Thymol | - | - | 2.1 | 0.7 | - | - | - | - | - | - |
| 32 | α-Cubebene | 0.1 | - | - | - | - | tr | - | 0.1 | 0.2 | 0.2 |
| 33 | Cyclosativene | 0.2 | - | - | - | - | - | - | 0.1 | - | 0.9 |
| 34 | α-Ylangene | 0.1 | - | 0.1 | - | - | - | - | - | - | 0.1 |
| 35 | α-Copaene | 0.7 | 0.3 | 1.6 | - | 0.2 | 0.6 | 0.3 | 0.5 | 0.7 | 2.4 |
| 36 | β-Cubebene | - | - | - | - | - | - | - | - | - | - |
| 37 | β-Elemene | 0.1 | 0.2 | - | 0.1 | 0.1 | 0.3 | 0.4 | - | 0.4 | 1.3 |
| 38 | Cyperene | 0.1 | 0.1 | - | - | - | 1.1 | 1.2 | 5.1 | - | - |
| 39 | α-Gurjunene | - | - | 1.3 | 0.1 | - | 0.1 | - | - | - | - |
| 40 | *cis*-α-Bergamotene | - | - | - | - | 0.2 | 0.3 | - | - | 0.7 | 0.8 |
| 41 | α-Santalene | - | 0.5 | - | - | 0.2 | 0.8 | - | - | 0.7 | - |
| 42 | (*E*)-β-Caryophyllene | 0.3 | 0.5 | - | 0.4 | 0.6 | 1.1 | 0.8 | - | 0.7 | 0.5 |
| 43 | β-Copaene | 0.1 | 0.5 | - | - | - | - | - | - | - | 3.0 |
| 44 | γ-Elemene | - | - | - | - | - | 1.0 | - | - | - | - |
| 45 | *trans*-α-Bergamotene | - | - | 0.2 | - | - | 0.2 | 0.2 | - | - | - |
| 46 | α-Guaiene | - | - | - | - | - | - | - | - | - | - |
| 47 | Aromadendrene | - | - | 0.2 | 0.9 | - | 1.1 | 0.3 | - | 0.8 | - |
| 48 | Spirolepechinene | - | - | - | - | - | - | - | 2.0 | - | - |
| 49 | (*E*)-β-Farnesene | - | - | - | - | - | - | - | - | - | - |
| 50 | α-Humulene | 0.1 | 0.1 | - | - | 0.1 | 0.4 | - | - | 0.2 | - |
| 51 | Rotundene | - | - | - | - | - | - | - | 4.2 | - | - |
| 52 | *allo*-Aromadendrene | - | 0.2 | 0.2 | 0.8 | 0.4 | 1.3 | - | - | 1.1 | - |
| 53 | Ishwarane | - | - | - | - | - | 0.7 | 0.2 | - | - | - |
| 54 | γ-Muurolene | - | - | - | - | - | - | - | - | 0.3 | - |
| 55 | Germacrene D | - | - | - | - | - | - | - | - | - | - |
| 56 | γ-Humulene | 0.3 | - | 1.0 | - | - | - | - | - | - | - |
| 57 | β-Selinene | 0.1 | 0.2 | - | 0.4 | 0.1 | 1.1 | 1.5 | - | 0.3 | 4.8 |
| 58 | α-Selinene | - | - | - | - | 0.2 | 0.8 | 0.8 | - | 0.3 | 1.9 |
| 59 | Isodaucene | - | - | - | - | - | - | - | - | - | - |
| 60 | α-Muurolene | 2.6 | 0.3 | - | - | - | - | 0.3 | - | - | 1.0 |
| 61 | (*E*),(*E*)-α-Farnesene | - | - | - | - | - | - | 0.1 | - | - | - |
| 62 | α-Bulnesene | - | - | - | - | - | - | - | - | - | - |
| 63 | β-Bisabolene | - | 0.3 | - | - | - | - | 1.0 | - | - | - |
| 64 | γ-Cadinene | 0.2 | 0.1 | - | - | 1.2 | 2.2 | 0.3 | - | 2.2 | 0.1 |
| 65 | *trans*-Calamenene | - | - | - | - | 0.1 | - | - | - | 1.6 | - |
| 66 | δ-Cadinene | 0.4 | 0.5 | 0.8 | 0.2 | 0.4 | 0.5 | 0.9 | - | 0.9 | 0.5 |
| 67 | α-Calacorene | - | - | - | - | - | - | - | - | - | - |
| 68 | Selina-4(15),7(11)-diene | - | - | - | - | - | 3.4 | - | - | 1.0 | - |
| 69 | β-Elemol | - | - | - | - | - | 0.6 | - | - | - | - |
| 70 | Selina-3,7(11)-diene | - | - | - | - | - | 2.0 | - | - | 0.4 | - |
| 71 | (*E*)-Nerolidol | 0.4 | 0.1 | - | - | 0.2 | 0.4 | 0.3 | - | - | - |
| 72 | Germacrène B | - | - | - | - | - | 1.3 | - | - | - | - |
| 73 | Spathulenol | - | - | - | 0.4 | - | 0.3 | - | - | 0.2 | - |
| 74 | Caryophyllene oxide | 0.2 | 0.1 | - | - | - | 0.8 | 0.5 | - | 0.2 | 0.3 |
| 75 | Viridiflorol | - | - | - | - | - | - | - | - | - | - |
| 76 | Ledol | - | - | - | 0.3 | - | 0.4 | - | - | - | - |
| 77 | Humulene oxide II | - | - | - | - | - | 0.2 | - | 0.1 | - | - |
| 78 | 1,10-di-*epi*-Cubenol | - | - | - | - | 0.2 | 0.2 | - | - | 0.2 | - |
| 79 | τ-Cadinol | 0.2 | 0.3 | - | - | 3.9 | 4.3 | 0.3 | - | 2.1 | - |
| 80 | τ-Muurolol | 0.3 | 0.1 | - | - | - | - | 0.3 | - | - | - |
| 81 | β-Eudesmol | - | - | - | - | - | - | - | - | - | - |
| 82 | α-Cadinol | - | - | - | - | - | - | 0.6 | - | - | - |
| 83 | α-Eudesmol | - | - | - | - | - | - | - | - | - | - |
|  |  |  |  |  |  |  |  |  |  |  |  |
|  |  | **92.6** | **92.6** | **97.6** | **90.5** | **98.0** | **86.3** | **90.9** | **85.3** | **89.7** | **83.3** |

Una: University Nangui Abrogoua; Mo: Montézo; Kd: Kodioussou; Ko: Kongofon.
